# PEGose Block Poly(lactic acid) Nanoparticles for Cargo Delivery

**DOI:** 10.1021/acs.macromol.4c00528

**Published:** 2024-06-14

**Authors:** Jean-Baptiste Masclef, Emmanuelle M. N. Acs, Jesko Koehnke, Joëlle Prunet, Bernhard V. K. J. Schmidt

**Affiliations:** † School of Chemistry, University of Glasgow, Joseph Black Building, G12 8QQ Glasgow, U.K.; ‡ Institute of Food Chemistry, Leibniz University Hannover, 30167 Hannover, Germany

## Abstract

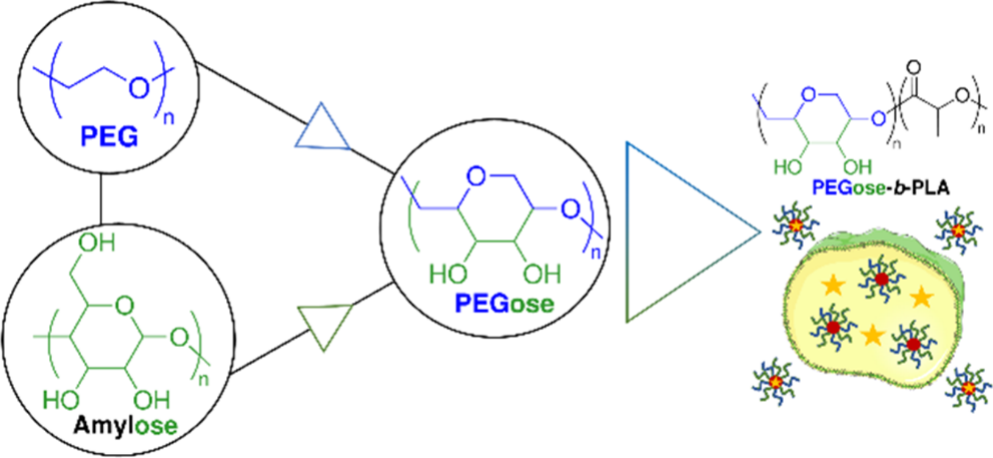

Hydrophilic polymers have found ubiquitous use in drug delivery and novel polymer materials to advance drug delivery systems are highly sought after. Herein, an amylose mimic (PEGose) was combined with poly(lactic acid) (PLA) in an amphiphilic block copolymer to form PEG-free nanoparticles as an alternative to PEG-based nanomedicines. The block copolymer self-assembled into 150–200 nm particles with a narrow dispersity in aqueous environment. The formed nanoparticles were capable of encapsulation, the sustained release of both hydrophilic and hydrophobic dyes. Moreover, the nanoparticles were found to be remarkably stable and had a very low cytotoxicity and a high propensity to penetrate cells. These results highlight the potential of PEGose-*b*-PLA to be used in drug delivery with a new hydrophilic building block.

## Introduction

Hydrophilic polymers play an important role in a broad range of drug-delivery systems.^1−3^ In particular, poly(ethylene glycol) (PEG) is employed frequently due to its favorable properties like biocompatibility and the stealth effect.^4^ Nevertheless, recent studies found anti-PEG antibodies that cause accelerated drug clearance.^5−8^ Other hydrophilic polymers showed promising results in drug-delivery systems as well, for example, poly(glycerol)s, poly(oxazoline)s, and poly(vinylpyrrolidone).^9−11^ Poly(glycerol)s are polyethers that share a close structural similarity with PEG, are also highly hydrophilic and biocompatible with a low viscosity in water.^12^ Poly(glycerol)s are also less susceptible to be degraded in response to thermal or oxidative stress.^13^ Poly(oxazoline)s, in particular poly(2-methyl-2-oxazoline) and poly(2-ethyl-2-oxazoline), have been successfully used as stealth polymers in drug delivery systems.^14−16^ Poly(vinylpyrrolidone)s seem like potent alternatives to PEG as they can enhance the blood circulation time of nanoparticles and display poor protein adsorption.^17−19^ Natural polymers like polysaccharides are also useful alternatives for PEG.^20,21^ Unlike PEG, polysaccharides are nonimmunogenic and can be functionalized in various ways. The additional benefit of polysaccharides is their anti-inflammatory and antioxidant effect, but most importantly, their ready biodegradability.^22,23^ Yet, this biodegradability imparts lower stability toward acids, bases, or enzymes. Moreover, they are usually obtained in a broad mixture of molecular weights, which hinders studies of structure–property relationships and introduces reproducibility issues. Additionally, their lack of solubility in most organic solvents makes them less easily functionalizable. Both synthetic and natural polymers have, therefore, numerous drawbacks. Other limitations include the lack of studies in clinical settings^24^ and the presence of some of these polymers in everyday products, leading to the apparition of antibodies after repeated use.^9,25^ Consequently, the search for hydrophilic polymers that might be employed in drug-delivery systems continues.

**Figure 1:**
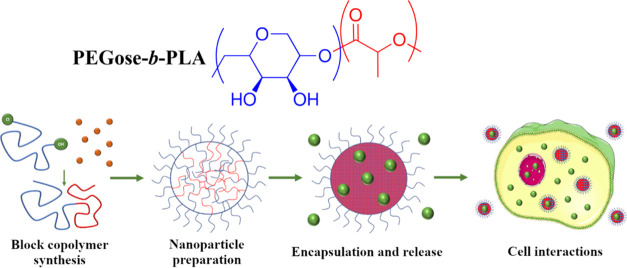
PEGose-*b*-PLA structure and schematic representation of its use as a drug carrier.

**Figure 2:**
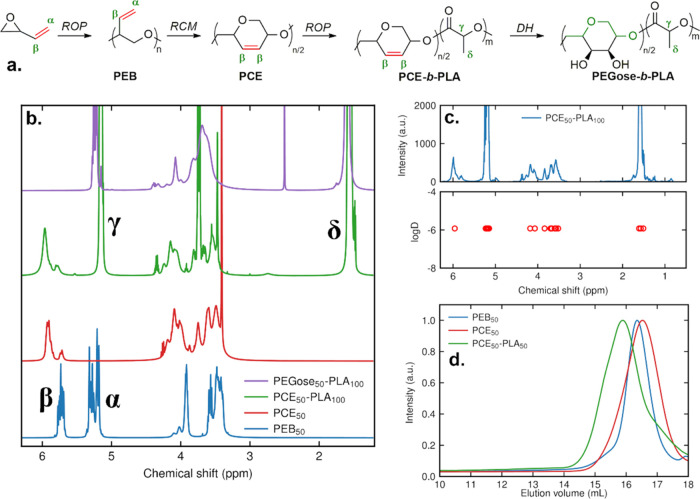
(a) Scheme of the synthesis of the PEGose-PLA block copolymer starting from butadiene monoxide. (b) ^1^H NMR spectra of the synthesized polymers in CDCl_3_ for PEB_50_, PCE_50_, and PCE_50_-PLA_100_ or DMSO-*d*_6_ for PEGose_50_-PLA_100_. (c) ^1^H DOSY-NMR spectra of the block copolymer PCE_50_-PLA_100_. (d) SEC traces of PEB_50_, PCE_50_, and all synthesized PCE–PLA block copolymers measured in THF.

In this regard, we recently reported the synthesis of a water-soluble amylose mimic coined PEGose in collaboration with the Shaver group.^26^ PEGose has a unique structure: it has both a polyether backbone like PEG and a polycyclic chain like amylose (Figure 1). The PEGose structure might be a convenient middle ground between synthetic polymers such as PEG or poly(glycerol)s, and natural polymers such as polysaccharides. It combines some advantages of these structures without some of their drawbacks. For example, PEG chains cannot be functionalized but end-groups are easily tuned. Polysaccharide chains can be conveniently functionalized, but it is difficult to only modify end-groups.^27,28^ PEGose precursor, on the other hand, is a functionalizable polycycloether that allows for a convenient modification of both the polymer backbone and its end-groups (see Figure 2a). Unlike common natural polysaccharides, PEGose mass and purity are easily controlled. A major difference between PEG and a polysaccharide is that PEG is a flexible polymer, while most polysaccharides, due to their polycyclic structure and their well-defined stereochemistry, are highly rigid.^29,30^ PEGose rigidity, however, can be determined by tuning its tacticity: atactic PEGose is amorphous and flexible; isotactic PEGose is helical and rigid. It is worth mentioning that unlike amylose, both helical handednesses can be readily obtained via monomer choice.^26^ Being stiffer than PEG, PEGose could form nanoparticles with a higher packing number, which may yield more stable nanoparticles and a better cargo retention.^31^

In order to form drug carriers, a common avenue is the formation of polymer particles from amphiphilic block copolymers.^32,33^ To prepare an amphiphilic copolymer, the choice of a hydrophobic block is equally important. Poly(lactic acid) (PLA) is a polymer widely used in the medical field, mainly due to its biocompatibility and biodegradability.^34−36^ PLA’s tacticity, like PEGose, can be easily tuned. It can be made atactic if dl-lactide is polymerized, or isotactic if l-lactide or d-lactide is used as the monomer. PLA is then either an amorphous or a semicrystalline polymer. PLA synthesis and its use as a nanoparticle core is extensively described in the literature.^37−44^ This work then aimed to produce a PEGose-*b*-PLA amphiphilic block copolymer as a PEG-free drug delivery system. Additional properties could also arise due to the novelty of the hydrophilic block used.

## Experimental Section

### Materials

Benzaldehyde, butadiene monoxide, calcium hydride, dl-lactide, *N*-methylmorpholine *N*-oxide, phenylboronic acid, potassium osmate, propionic acid, pyrrole, Sephadex LH-20, and stannous octoate were obtained from Alfa Aesar. Diethylaluminum chloride solution in hexane (1 M), fluorescein isothiocyanate-labeled bovine serum albumin, and rhodamine B were obtained from Sigma-Aldrich. Grubbs II catalyst was purchased from Carbosynth. Deuterium oxide was purchased from Fluorochem. Tetrahydrofuran, dichloromethane, and toluene were obtained using an in-house solvent purification system (Pure-SolvTM 500 Solvent Purification System). Other solvents were purchased from Fisher Chemicals and were of HPLC grade. Gibco Minimum Essential Medium (MEM), Gibco fetal bovine serum (FBS), Gibco 0.25% trypsin-EDTA (1×), Gibco MEM nonessential amino acid solution (100×) (without l-glutamine), Gibco l-glutamine solution (200 mM), Gibco penicillin-streptomycin (with 10,000 units penicillin and 10 mg streptomycin/mL), and Gibco Phosphate Buffered Saline (10×) (PBS) solution were purchased from Fisher Scientific. AlamarBlue Cell Viability Reagent and Prolong Antifade Glass mountant were purchased from Invitrogen. 4% Formaldehyde in PBS was purchased from ChemCruz. 96-Well cell culture microplates were obtained from Corning. Cell-culture-treated Nunc Lab-Tek II Chamber Slide systems for microscopy sample preparation were purchased from Thermo Fisher Scientific.

Butadiene monoxide was dried over calcium hydride for 24 h under reflux and distilled under an atmosphere of argon. dl-Lactide was purified by recrystallization from ethanol, followed by another recrystallization from ethyl acetate. Reactions involving air-sensitive agents and dry solvents were performed in glassware that had been oven-dried (150 °C) or flame-dried prior to use. These reactions were carried out with the exclusion of air using an argon atmosphere. Preparative size exclusion chromatography was performed under forced flow conditions using HPLC-grade solvents and Sephadex LH-20 as solid support. Tetraphenylporphyrin was synthesized according to the literature procedure.^26^

### Methods

#### Block Copolymer Synthesis

##### Synthesis of Poly(epoxybutene)—PEB_50_

PEB was synthesized by a modified literature procedure.^26^ Tetraphenylporphyrin aluminum chloride TPPAlCl was prepared in a flame-dried Schlenk tube by dissolving tetraphenylporphyrin (1.35 g, 2.20 mmol, 1 equiv) in dichloromethane (40 mL) and slowly adding a 1 M diethylaluminum chloride solution in hexane (2.20 mL, 1 equiv). After 3 h, the volatiles were evaporated using vacuum and a liquid N_2_-cooled trap. TPPAlCl was dried overnight. Racemic 3,4-epoxy-1-butene (14.0 g, 22.0 mmol, 100 equiv) was added the next day to the dried TPPAlCl, and the resulting mixture was subsequently stirred at ambient temperature for 2 days. The reaction was quenched with a few drops of 1 M aqueous HCl under stirring. The volatiles were removed, and the resulting polymer was dissolved in a 1:1 methanol/dichloromethane mixture for purification by size exclusion chromatography (Sephadex LH-20, 1:1 methanol/dichloromethane). The fractions were concentrated, and the polymer was dried under vacuum overnight to give a brown oil (11.5 g, 82%).

##### Synthesis of Polycycloether—PCE_50_

PCE was synthesized by a modified literature procedure.^26^ In a large round-bottom flask, PEB (600 mg, 0.25 M) was stirred for 15 min at 84 °C in 1,2-dichloroethane (38 mL). Then, second-generation Grubbs catalyst (178 mg, 210 μmols, 2.5 mol %) in dichloroethane (5 mL) was added slowly under argon. After 5 days, the reaction mixture was cooled to ambient temperature, 1.5 mL (100 equiv) of dimethyl sulfoxide (DMSO) was added, and the mixture was stirred for 1 h. The volatiles were removed, and the residue was purified using size exclusion chromatography (Sephadex LH-20, 3:1 methanol/dichloromethane). The volatiles were removed, and 5 mL of dichloromethane was added. Adapted from a literature procedure,^45^ the mixture was cooled in an ice bath, 2.5 mL (100 equiv) of 30% aqueous hydrogen peroxide was added and vigorously stirred for 1 h. The layers were then separated, and the organic phase was washed with 50 mL of an aqueous sodium sulfite solution (100 mg/mL). The oxidative process was repeated one more time, the volatiles were removed, and the resulting PCE was dried overnight to obtain a gray solid (198 mg, 52%).

##### Synthesis of Polycycloether Block Poly(lactic acid)—PCE_50_-*b*-PLA_100_

To synthesize PCE_50_-*b*-PLA_100_, pure dl-lactide (300 mg, 1.04 mmol, 2 equiv) and PCE (100 mg, 0.520 mmol, 1 equiv) were mixed in anhydrous toluene (5 mL), and the mixture was degassed by the freeze–pump–thaw method. Under an argon atmosphere, stannous 2-ethyl-hexanoate (5 mg, 12 μmols, 0.01 equiv) was added, and the reaction mixture was refluxed in an oil bath for 3 h. Subsequently, the toluene was removed under reduced pressure, and the residue was solubilized in tetrahydrofuran (THF) to perform purification by size exclusion chromatography (Sephadex LH-20, THF) to obtain PCE-*b*-PLA as a brown oil (348 mg, 87%).

##### Synthesis of PEGose Block Poly(lactic acid)—PEGose_50_-*b*-PLA_100_

To synthesize PEGose_50_-PLA_100_, PCE-*b*-PLA (448 mg, 1.0 mmol of the PCE monomer unit) was dissolved in acetone/water (5:1, 20 mL). Dried *N*-methylmorpholine-*N*-oxide was then added to the mixture (129 mg, 1.1 mmol, 1.1 equiv). The reaction mixture was stirred in an ice bath, and then 0.1 mol % of freshly prepared OsO_4_ solution (100 μL, 1% in water) was added slowly, and the resulting mixture was stirred for an additional 2 h. The reaction mixture was stirred overnight at ambient temperature. To quench the reaction, 126 mg (1 equiv) of sodium sulfite was added and the resulting mixture was stirred for 1 h. Water was added (20 mL) and the mixture was then dialyzed (3.5 kDa molecular weight cutoff (MWCO)) against deionized water over 24 h, regularly changing the water. The resulting solution was evaporated to yield PEGose-*b*-PLA as a gray solid (357 mg, 74%).

##### Synthesis of Diborated PCE

Diborated PCE synthesis was adapted from Nóvoa et al.^46^ In a flame-dried round-bottom flask, PCE (112 mg, 0.5 mmol, 1 equiv), B_2_pin_2_ (254 mg, 1.0 mmol, 2 equiv), and NaOMe (32 mg, 0.3 mmol, 0.6 equiv) were dissolved in a minimum amount of methanol, and the resulting solution was refluxed overnight. The mixture was purified by SEC (Sephadex LH-20, methanol) and the volatiles were removed under vacuum to afford the diborated PCE as a brown oil (300 mg, 82%).

##### Synthesis of Osmium-Free PEGose

Adapted from a literature procedure,^46^ diborated PCE (100 mg, 0.27 mmol, 1 equiv) and aqueous 1 M NaOH (2.7 mL, 2.7 mmol, 10 equiv) were mixed. Then, a 30% solution of H_2_O_2_ (0.3 mL, 5.4 mmol, 20 equiv) was added dropwise. The mixture was stirred overnight at 80 °C. The mixture was dialyzed (3.5 kDa of MWCO) against deionized water overnight. The volatiles were evaporated to obtain osmium-free PEGose as a gray solid (13 mg, 34%).

##### Synthesis of Fluorescein Isothiocyanate-Labeled PEGose

Adapted from de Belder and Granath procedure,^47^ fluorescein isothiocyanate-labeled PEGose (FITC-PEGose) and PEGose-*b*-PLA were prepared by mixing the polymer (100 mg) in 1 mL of dry DMSO and 50 μL of pyridine. The fluorescent dye isothiocyanate fluorescein was then added (10 mg), followed by dibutyltin dilaurate (2 mg). The reaction mixture was heated to 95 °C for 2 h. Subsequently, the labeled polymer was dialyzed (3.5 kDa MWCO) against deionized water over 48 h, regularly changing the water. The water solution was then concentrated and the polymer dried *in vacuo* (84 mg, 84%, d.s. 0.0005). The degree of substitution was determined by preparing a standard curve with FITC solutions in a Tris buffer, with concentrations ranging from 10^–6^ to 10^–5^ mol/L. The wavelength of absorption was fixed to 493 nm and labeled polymers were dissolved in a Tris buffer.

#### Nanoparticle Preparation

Typical procedure to obtain 100–150 nm PEGose-PLA nanoparticles: PEGose-*b*-PLA block copolymer was dissolved in DMSO (0.6 mL) with a concentration of 10 mg/mL and then 2 mL of deionized water was added dropwise slowly under stirring (300 rpm) using an MS-H280-Pro hot plate stirrer. The drop rate was kept relatively constant, by hand, using a syringe, over approximately 2 min. The solution was then dialyzed overnight against an excess of deionized water to remove the DMSO (3.5 kDa MWCO).

#### Dye Loading

Rhodamine B or Nile Red was solubilized in a 5 mg/mL PEGose-*b*-PLA block copolymer solution in DMSO, and 2 mL of deionized water was added dropwise. The Rhodamine B solution was dialyzed against a 1000-fold excess of deionized water for 4 h; the water was changed after 30 min, then every hour. Nile Red was dialyzed against a 1000-fold excess of deionized water for 4 h and then filtered to remove insoluble residues.

### Characterization

^**1**^**H NMR and DOSY** spectra were recorded on either a Bruker AVI DPX-400 or a Bruker DPX-400 (400 MHz) instrument. The chemical shifts are expressed in parts per million (ppm) referenced to TMS. CDCl_3_ was used as the solvent except for PEGose-*b*-PLA where DMSO-*d*_6_ was used. Size exclusion chromatography (SEC) was conducted in THF at 35 °C using a column system with an Agilent PL Gel Guard Column (5 μm) and an Agilent PL Gel Mixed-D Column (5 μm) as well as an Agilent Infinity1260 II RID and calibration with poly(styrene) standards. **Dynamic light scattering (DLS)** was performed on a Zetasizer by Malvern with HPLC-grade water as the solvent. All experiments were performed three times using backscattering (173°) and average size distribution was calculated. **ζ Potential** measurements were performed on a Zetasizer by Malvern with HPLC-grade water as the solvent, using DTS1070 disposable cuvettes. Experiments were performed 10 times; data quality was checked using the ZS Xplorer software. **Transmission electron microscopy (TEM)** experiments were performed on a JEOL 1200 EX TEM running at 80 kV; tiff images were captured using a Cantega 2K X 2K camera and an Olympus ITEM Software. To prepare negative stained samples: suspension droplets (5 μL) were placed on top of the surface of carbon-coated 400 mesh copper grids which were previously glow-discharged using a Quorum Q150T ES high-vacuum system. Samples were left for 5 min to allow attachment, then grids were floated sample side down three times for 30 s each onto distilled water droplets before negative staining with 2% aqueous uranyl acetate for 5 min then allowed to air-dry. To prepare unstained samples, droplets (5 μL) were placed on top of the surface of carbon-coated 400 mesh copper grids, which were previously glow-discharged using a Quorum Q150T ES high-vacuum system. Sample grids were left to air-dry before digital imaging. **High-resolution mass spectrometry (HRMS)** was performed on a Bruker microTOFq high-resolution mass spectrometer using an electrospray (ESI) ion source coupled to a time-of-flight (ToF) analyzer. **Multiangle dynamic light scattering (MADLS)** measurements were performed using an Anton Paar Litesizer 500 using forward scattering (15°), side scattering (90°), and backscattering (175°). The light source was a semiconductor laser diode at 40 mW, 658 nm. **Inductively coupled plasma mass spectrometry (ICP-MS)** experiments were made on an Agilent 7500ce ICP-MS fitted with a self-aspire Teflon nebulizer doing 10 repeats per peak on masses 101, 102, and 104. Concentrations were calculated against the Ru standard Alfa Aesar Specpure. **Fluorescence spectroscopy** studies were performed on a Horiba Duetta Bio fluorescence and absorbance spectrometer. A blank was used before each experiment. Both blank and samples absorption spectra were recorded before each experiment to correct the inner filter effect. Excitation wavelength was fixed at 435 nm, and emission wavelength was recorded from 445 to 800 nm. Integration time was 0.1 s, and detector binning was 0.5 nm (1 pixel). Excitation and emission bandpass were 5 nm. **UV–vis spectroscopy** measurements were performed using a Shimadzu UV Mini 1240 UV–vis spectrophotometer at ambient temperature. To calculate **Rhodamine B encapsulation efficiency** and characterize its successful loading in the nanoparticles, the unloaded dye was removed through dialysis, and the absorbance was measured through a UV–vis spectrometer. An empty nanoparticle solution was used as a blank. The time needed to remove all unloaded dyes was determined by doing the dialysis of a solution of pure Rhodamine B in water and measuring the time needed to reach a near-zero absorbance.
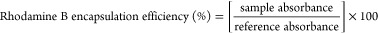
**Rhodamine B release profiles** were measured by keeping empty and loaded nanoparticle solutions in a 10 kDa MWCO dialysis bag with a 1000-fold excess volume of deionized water, changing the water twice a day. The absorbance was measured and compared to an empty nanoparticle solution as a reference.

**Nile Red encapsulation efficiency** was determined after removing insoluble residues, the water solution was evaporated, solubilized in DMSO sonicated for 15 min, and then stirred overnight. Fluorescence intensity was then measured and compared to the Nile Red DMSO reference solution.^48^
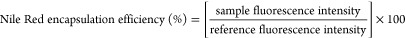
**Nile Red release profile** was determined by measuring the fluorescence intensity of the samples over time and comparing it to the fluorescence intensity just after removing unloaded Nile Red.
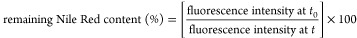


### Nanoparticle Stability Tests^49^

PEGose-*b*-PLA nanoparticles were prepared in deionized water, dialyzed as previously described, and then transferred in a variety of buffers to evaluate their stability. Buffers, enzyme, or salts were added to obtain five different conditions: deionized water at ambient temperature (control sample), deionized water at 37 °C, 0.1 M Tris with 0.17 mg/mL proteinase K from *Tritirachium album* at 37 °C, deionized water with 10 mg/mL sodium cholate hydrate at 37 °C, pH 5.5 solution prepared from diluted HCl at 37 °C. Nanoparticle size distributions were monitored every day for 4 days using DLS. Additionally, for the proteinase K solution, a qualitative *p*-hydroxydiphenyl test was realized to check the presence of lactic acid or PLA oligomers. Briefly, one drop of the nanoparticle solution was taken and 1 mL of concentrated sulfuric acid was added. The solution was heated to 85 °C then cooled down to ambient temperature. After cooling down, a pinch of solid *p*-hydroxydiphenyl was added and the mixture was stirred for 10 min. Finally, the color of the solution was inspected. A purple color would indicate the presence of lactic acid or PLA oligomers.

### Protein Aggregation Test^50^

PEGose_50_-PLA_50_ and PLA_100_ nanoparticle solutions were prepared in PBS (15 mM, pH 7.4) at a concentration of 2 mg/mL following a previously described nanoparticle preparation procedure. Fluorescein isothiocyanate-labeled bovine serum albumin (FITC-BSA) was added in both solutions to obtain a 100 μg/mL concentration of protein. The two solutions were kept at 37 °C for 24 h and then centrifugated at 20,000 g for 20 min. The fluorescence intensity of the supernatant was measured at a 490 nm wavelength and compared to the fluorescence intensity of a 100 μg/mL FITC-BSA solution.

### Biocompatibility^51^

Human hepatocellular carcinoma cell line (HepG2, ECACC No. 85011430) was cultured in MEM medium supplemented with 10% FBS, 100 U/mL penicillin, 100 μg/mL streptomycin, 2 mM l-glutamine, and 1% nonessential amino acids. All cells were grown in a humidified incubator at 37 °C under 5% CO_2_ and passaged every 3 days.

Cells were seeded at a density of 10^4^ cells/well into 96-well plates and incubated at 37 °C in a 5% CO_2_ atmosphere. The second day, the cells were treated with the different samples (a-PEGose and PEGose-*b*-PLA) at 0.5, 5, 50, and 500 μg/mL and incubated for 24 h at 37 °C in 5% CO_2_. After the incubation period, AlamarBlue reagent was added to each well according to the manufacturer’s instructions (10% v/v) and cells were incubated at 37 °C in 5% CO_2_ for an additional 4 h. The fluorescence measurements were performed by using a CLARIOstar microplate reader (BMG Labtech, Ortenberg, Germany) (λ_exc_ = 560 nm; λ_em_ = 590 nm). The percent cell viability was calculated in reference to the untreated control cells using the following formula:
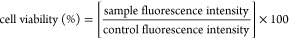
Cells without treatment with samples were considered as the control. The percent cell viability was calculated by comparing the fluorescence intensities of each well to the fluorescence intensity of the untreated cells. This assay was repeated 3 times on different passages of cells. The median value of the cell viability was selected for graphical representation.

### Cell Permeation Experiment and Confocal Laser Scanning Microscopy (CLSM)^52^

FITC-labeled (emission wavelength λ = 493 nm) samples (PEGose homopolymer and PEGose_50_-PLA_50_ nanoparticles) were dissolved in water (10 mg/mL). Samples for CLSM were prepared by seeding 2 × 10^4^ cells in each well of the Nunc Lab-Tek II Chamber Slide as well as treated with polymer samples on the same day (final conc. 50 μL/mL). Cells without treatment with polymer samples were considered as a control. Slides were incubated at 37 °C in a 5% CO_2_ atmosphere for 24 h. The second day, the cells were washed with PBS (1%) and then fixed with 4% formaldehyde in PBS for 15 min before being washed twice with PBS (1%). The samples were layered with an antifade mountant and a thin cover glass. Slides were left to dry and covered in a laminar flow cabinet overnight before being stored in the dark at 4 °C.

CLSM and bright-field microscopy were performed on a Zeiss LSM710 confocal microscope (Zeiss, Göttingen, Germany) using Carl Zeiss ZEN 2011 v7.0.3.286. 0.55 DIC (Carl Zeiss, White Plains, NY), Neofluar 20×, and N-Achroplan 10 × /0.25 Ph 1 (Carl Zeiss, White Plains, NY) objectives were used. The images were taken with two different channels: one for the fluorescent polymers (FITC, 486–570 nm) and one for a bright-field image of the cells.

## Results and Discussion

### Block Copolymer Synthesis

#### PEB Synthesis

Poly(epoxybutene) (PEB) was synthesized by the ring-opening polymerization of commercially available butadiene monoxide (Figure 2a). The reaction was performed in a Schlenk tube, under inert atmosphere and at ambient temperature employing tetraphenylporphyrin aluminum chloride (TPPAlCl) as a catalyst and initiator, which was synthesized according to the literature in a 2-step procedure.^26^ A 1:50 ratio of butadiene monoxide/TPPAlCl was used to obtain a polymer with approximately 50 repeating units, yielding a number average molar mass of 3.5 kDa after 3 days, with a narrow dispersity (*Đ* < 1.20) according to SEC (See Table 1). The polymer structure was determined by ^1^H NMR, the two terminal protons from the vinyl group displayed a peak around 5.2 ppm, the remaining alkene proton showed up at 5.7 ppm, the proton from the carbon bearing the vinyl group showed up at 3.9 ppm and the two remaining protons showed up as a multiplet between 3.6 and 3.4 ppm (See Figure 2). The polymer end-groups, a hydroxy group and chlorine atom, were confirmed by mass spectrometry using electrospray ionization and a low mass PEB (Figure S1). The hydroxy end-group was exploited later as an initiator for the ring-opening polymerization of dl-lactide.

**Table 1:** Theoretical Polymer Masses and Experimental Polymer Masses Measured by SEC and ^1^H NMR

**polymer**	***M***_**n**_**SEC**^a^**(kDa)**	***M***_**n**_**NMR**^b^**(kDa)**	***M***_**n**_**Theo**^c^**(kDa)**	***Đ***^a^
PEB_50_	3.5		3.6	1.27
PCE_50_	2.9		2.8	1.37
PCE_50_-PLA_15_	4.7	3.7	3.9	1.34
PCE_50_-PLA_25_	5.0	4.8	4.6	1.32
PCE_50_-PLA_50_	5.6	6.3	6.4	1.37
PCE_50_-PLA_100_	7.1	10.9	10.0	1.52

a measured via SEC in THF with a 1 mL/min flow rate.

b measured via ^1^H NMR in CDCl_3_.

c calculated from starting material equivalents

#### Ring-Closing Metathesis

In the next step, a functionalizable polycycloether (PCE) was synthesized by performing a ring-closing metathesis on PEB. Compared to the previous PCE synthesis described in the literature,^26^ Grubbs II catalyst was used with a decreased amount of 2.5 mol %. Since the synthesized polymer is meant to be used for drug delivery, the main challenge of the block copolymer synthesis was to avoid any residual metals to achieve a high biocompatibility. Thus, residual ruthenium was removed employing an oxidative procedure described previously.^45^ The conversion of PEB to PCE was monitored by integrating the terminal alkene proton peak at 5.3 ppm (α proton, see Figure 2b) corresponding to the PEB starting material with the internal alkene proton peak at 5.7 ppm (β proton, see Figure 2b). Even though a conversion of 80% could be achieved in an hour, nearly full conversion (>99%) could only be achieved after 5 days. The number average molar mass of the resulting polymer was 2.9 kDa according to SEC, which supports the elimination of ethylene during the ring-closing metathesis reaction. Ruthenium content before and after various purification methods was quantitatively evaluated by ICP-MS. Results showed that 99.5% of the ruthenium was removed successfully with the oxidative procedure (see Table S1).

#### PCE-*b*-PLA Block Copolymer Synthesis

The diblock copolymer PCE-*b*-PLA was synthesized by ring-opening polymerization of dl-lactide using PCE as an initiator and stannous octoate as a catalyst.^53^ The reaction was performed under an inert atmosphere in toluene under reflux for 3 h. Four block copolymers were synthesized comprising four different PLA lengths (15, 25, 50, and 100 repeating units) with a fixed PEGose length of 25 repeating units. It should be noted that the number of repeating units halves after RCM from PEB to PCE. To be consistent, we kept the subscript of PCE the same as for the PEB block. PCE-*b*-PLA block copolymer structure was confirmed by ^1^H NMR, DOSY-NMR, and SEC. ^1^H NMR showed peaks corresponding to both blocks, e.g., 5.2 and 1.2 ppm for the PLA block and 5.8 and 4.0–3.5 ppm for the PCE block (Figure 2b). Furthermore, DOSY-NMR showed that PCE and PLA peaks had the same diffusion coefficient regardless of the PCE/lactide ratio used for the ring-opening polymerization (Figure 2c), thus confirming the block copolymer structure. SEC-derived number average molar masses of the block copolymers were slightly lower than expected for PCE_50_-PLA_50_ and PCE_50_-PLA_100_ (see Table 1), which might be due to the structural difference between the two blocks and the poly(styrene) standards used for the calibration curve. Nevertheless, the shift of the peak after polymerization with decreasing PCE/lactide ratios confirmed the chain extension of the polymer. Looking at the SEC elution trace, a single peak could be observed, suggesting that no PLA homopolymers were formed or that the Sephadex purification step successfully removed any PLA homopolymers (see Figure 2). The mass of the block copolymers was also determined by integrating ^1^H NMR peaks from PLA against peaks from PCE. This method yielded masses similar to the theoretical ones calculated from the PCE/lactide ratio (see Table 1). While the block copolymer dispersities were quite high considering the polymerization type (*Đ* = 1.32 for PEGose_50_-PLA_25_ up to *Đ* = 1.52 for PEGose_50_-PLA_100_), a recent study showed that a high polymer dispersity might be advantageous to obtain nanoparticles with a narrow size distribution.^54^

#### PEGose-*b*-PLA Synthesis

The dihydroxylation of the PCE alkene was achieved by using catalytic osmium tetroxide and *N*-methylmorpholine *N*-oxide at ambient temperature in a water/acetone solvent mixture. The conversion was monitored by ^1^H NMR in DMSO-*d*_6_ by integrating the alkene peak at 5.7 ppm. Full conversion was achieved overnight. Fully converted PEGose-PLA was found to be only soluble in DMSO. Due to the insolubility of PEGose-PLA in most organic solvents, the number average molar mass of the block copolymer was calculated from the number average molar mass of PCE–PLA. In order to achieve a high biocompatibility, another route was developed to avoid the use of osmium. First, the diboration of PCE with *bis*(pinacolato)diboron was performed, followed by the addition of hydrogen peroxide and sodium hydroxide to obtain an osmium-free PEGose. However, this method gave a lower yield and conversion, while not significantly improving the biocompatibility of the polymer (Figure 6).

### Nanoparticle Preparation

**Figure 3:**
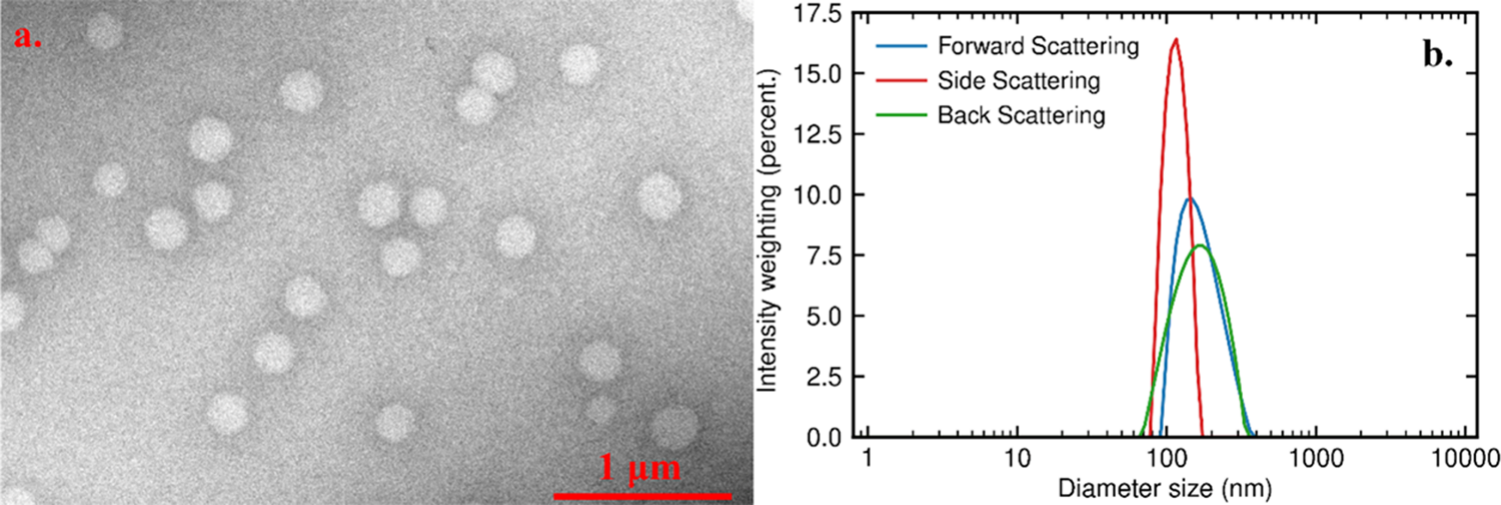
(a) TEM image of PEGose_50_-PLA_25_ nanoparticles without using any stain. (b) MADLS size measurements of PEGose_50_-PLA_25_ nanoparticle dispersion in deionized water (0.5 mg/mL, 25 °C) at 15, 90, and 175°.

In order to form nanoparticles, the solvent-switch method was used.^55−59^ This process generally yields porous nanoparticles instead of micelles. The average diameter of the nanoparticles was determined by using multiangle dynamic light scattering (MADLS) (see Figure 3). The hydrodynamic diameter size ranged between 150 and 200 nm. No clear correlation could be observed between the size of the nanoparticles and the length of the PLA chain (Table 2). A very slow addition of water in the copolymer DMSO solution was found to be key to obtain reproducible results with a low dispersity. The size of the nanoparticles tended to increase over time, presumably due to aggregation. Thus, ζ potential measurements were performed on PEGose_50_-*b*-PLA_50_ and PEGose_50_-*b*-PLA_100_. PEGose_50_-*b*-PLA_100_ nanoparticles dispersion had a ζ potential of −5.86 mV, while PEGose_50_-*b*-PLA_50_ had a ζ potential of −4.20 mV. These values are similar to the ζ potential values reported for PEG-*b*-PLA nanoparticles, usually ranging between −2.0 and −11.0 mV.^52,60^ The low absolute value of the ζ potential (less than 30 mV) might be a reason for the aggregation over time of the nanoparticles. However, extruding the nanoparticle solution through a 0.2 μm filter yielded nanoparticles with the same size as when they were initially formed.

**Table 2:** Hydrodynamic Diameter of the Nanoparticles in Deionized Water (0.5 mg/mL) at 25 °C Prepared from PEGose_50_-PLA_100_, PEGose_50_-PLA_50_, PEGose_50_-PLA_25_, and PEGose_50_-PLA_15_, Measured by MADLS Using Intensity Weighting

**PLA units**	**hydrodynamic diameter (nm)**	**polydispersity index**	**measurement angle (deg)**	**standard deviation (nm)**
25	149	0.25	15	2.6
25	156	0.03	90	1.8
25	164	0.20	175	3.5
100	190	0.21	175	1.8
50	192	0.16	175	2.1
15	192	0.22	175	2.4

**Figure 4:**
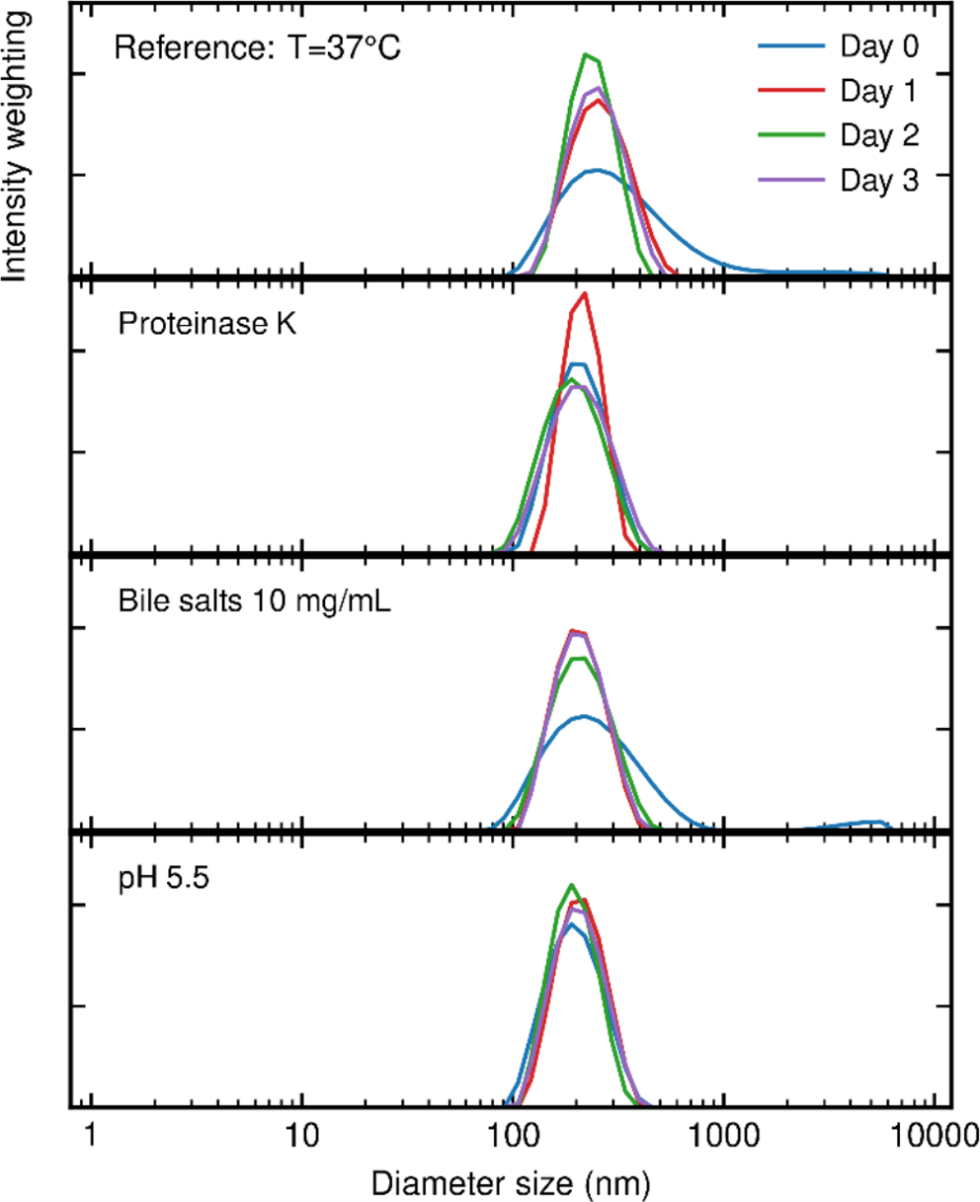
DLS size distribution with intensity weighting of a PEGose_50_-PLA_50_ nanoparticle dispersion (0.5 mg/mL) over 4 days at 37 °C, under different conditions: (a) deionized water, (b) 0.1 M Tris with 0.17 mg/mL proteinase K from Tritirachium Album, (c) 10 mg/mL sodium cholate hydrate in deionized water, and (d) pH 5.5 solution prepared from diluted HCl.

**Figure 5:**
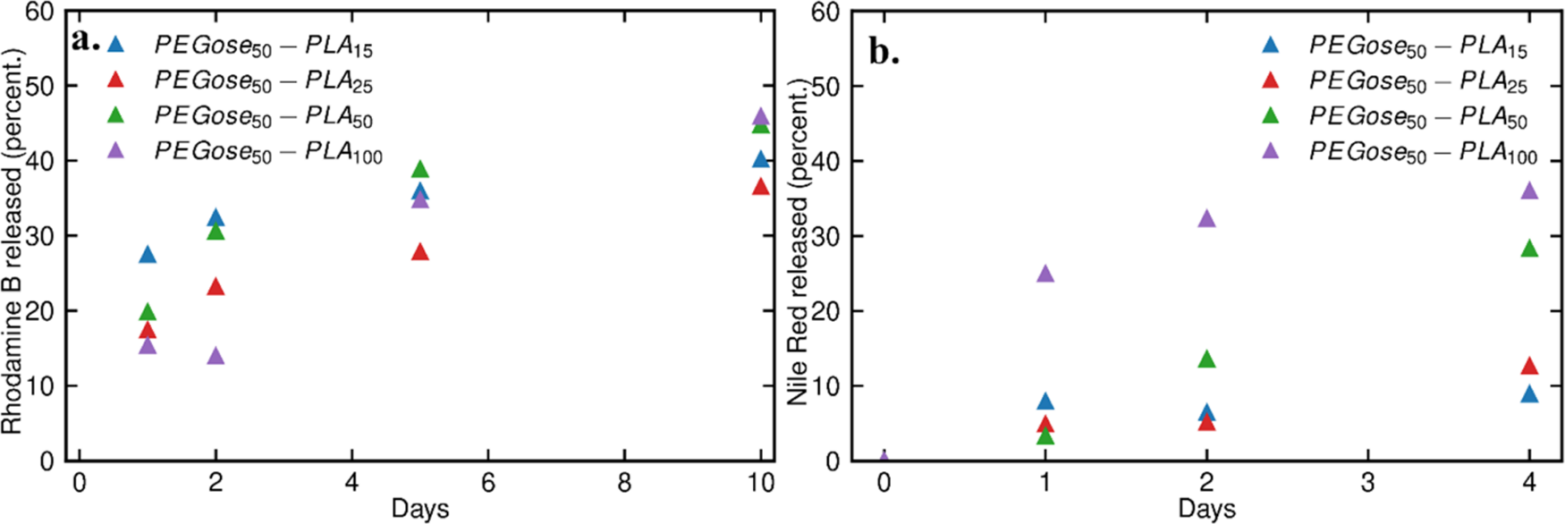
(a) Amount of Rhodamine B released over 10 days measured by UV–vis spectroscopy. (b) Amount of Nile Red released over 4 days measured by fluorescence spectroscopy.

While TEM confirmed the spherical structure of the nanoparticles and their size (Figure S4), it did not give any insight into the internal structure of the nanoparticles (Figure 4). Based on the low molecular weight of the block copolymers (between 5 and 11 kDa), the nanoparticles cannot be traditional polymer micelles. Moreover, both negative staining with uranyl acetate and the absence of any staining supplied images that did not indicate any hollow architecture (Figure S5). To further prove the absence of a core–shell/hollow structure, the nanoparticles’ response toward an osmotic shock was studied. Nanoparticles were formed in deionized water, with a hydrophilic dye (Rhodamine B) encapsulated and then added to a 100 mg/mL NaCl in water solution. While the size of the nanoparticles increased, it did not lead to any cargo release (see Figure S3), which would be expected for vesicles.^61^ Another argument invalidating the traditional polymer micelle structure is the ability of PEGose-PLA NPs to encapsulate hydrophilic compounds such as the hydrophilic dye Rhodamine B (Figure 5). A single-layer polymersome/vesicle structure is also unlikely due to the very high contrast obtained with electron microscopy even without staining (Figure 3a). For PEGose being less hydrophilic than PEG, a stronger interaction between the chains is expected, which probably led to these highly dense nanoparticles. As these nanoparticles are neither micelles nor vesicles/polymersomes, we assume that complex aggregates are formed with a PEGose corona and a mixed core composed of PEGose-PLA aggregates. The chain packing parameter theory could also explain why no micelles were obtained; PEGose is more rigid than PEG, a decreased chain mobility leads to a decreased hydrophilic volume/surface, thus a higher packing parameter, producing more complex structures.

### Nanoparticle Stability Tests

Because of the highly packed structure of the nanoparticles, we envisaged an increased stability compared to that of regular micelles and vesicles. To investigate the stability of these particles in different media, their size was monitored over several days by using DLS. The nanoparticles’ size remained stable over 3 days at 20 or 37 °C (Table 3). The nanoparticles even continued to be stable over 1 year when stored at 4 °C after extrusion through a 0.2 μm filter (see Table S2). The PEGose corona proved to be able to prevent the degradation of PLA by the Proteinase K enzyme, which has been employed to degrade PLA before.^62^ Additionally, a *p*-hydroxydiphenyl test was realized to detect the presence of lactic acid or PLA oligomers, but the presence of neither species could be found. The PEGose corona also prevented the adsorption of FITC-labeled bovine serum albumin (FITC-BSA) proteins (see Figure S11). Briefly, FITC-labeled BSA proteins were incubated with either PEGose_50_-PLA_50_ or PLA_100_ nanoparticles. The nanoparticles were then centrifugated, and the fluorescence intensity was measured to determine how much of the protein adsorbed on the nanoparticles. Almost no protein adsorbed on the PEGose-PLA nanoparticles, while approximately half of the proteins adsorbed on the PLA nanoparticles, which confirmed the potential shielding effect of the PEGose corona. A bile salt concentration of 10 mg/mL and a pH of 5.5 that can be found in the gastrointestinal tract of the human body^63^ did not have any impact on the size of the nanoparticles as well. The ability of the PEGose-PLA nanoparticles to withstand enzymes and bile salts and low pH at 37 °C could be useful for oral delivery, where highly stable drug delivery systems are needed. Targeting the gastrointestinal tract could lead to promising results, as traditional vesicular structures such as liposomes or polymersomes struggle to resist this harsh environment.^64^

**Table 3:** Encapsulation Efficiencies of Rhodamine B and Nile Red for All Four Copolymers Synthesized: PEGose_50_-PLA_100_, PEGose_50_-PLA_50_, PEGose_50_-PLA_25_, and PEGose_50_-PLA_15_

**dye encapsulated**	**PLA units**	**encapsulation efficiency (%)**
Rhodamine B	100	49
	50	68
	25	34
	15	14
Nile Red	100	14
	50	52
	25	49
	15	41

### Dye Loading and Release

To provide an alternative to vesicle formulations, a drug delivery system should be able to encapsulate both hydrophobic and hydrophilic compounds. Here, the encapsulation and release of two different dyes were studied by using four different PEGose-PLA block copolymers. While the length of the PLA chain did not have an impact on the size of the nanoparticles (See Table 2), it had an impact on the encapsulation of both the hydrophobic dye Nile Red and the hydrophilic dye Rhodamine B (see Figure 5). The highest encapsulation efficiency for both Rhodamine B and Nile Red was achieved by using PEGose_50_-PLA_50_. For Rhodamine B, 68% of the dye was successfully encapsulated, 52% in the case of Nile Red. Interestingly, for Nile Red, there was a sharp decrease in the encapsulation efficiency when increasing the PLA length from 50 units to 100 units. Similarly, for Rhodamine B, the encapsulation efficiency declined to 14% for PEGose_50_-PLA_15_. These results illustrate the fact that a longer hydrophobic chain does not systematically lead to better encapsulation of hydrophobic compounds. A similar phenomenon was described by Gianneschi and co-workers,^65^ who deduced the mismatch between chain length and encapsulation efficiency of hydrophobic compounds to the lower mobility of longer hydrophobic blocks in the micelle core and during micelle formation. In our study, the opposite effect was found for the correlation of hydrophobic chain length and encapsulation of hydrophilic compounds, as elongation of the hydrophobic block led to an increase of hydrophilic dye encapsulation. This can most likely be explained by the decreasing mobility of hydrophilic PEGose chains at the interface of particles and even more so for internalized PEGose chains anchored to longer PLA with increasing hydrophobicity. Thus, both PEGose and PLA blocks play an important role in the encapsulation of hydrophobic and hydrophilic dyes.

The release of both dyes was measured by fluorescence or UV–vis spectroscopy for Nile Red and Rhodamine B in deionized water, respectively. In most cases, the nanoparticles released some of their cargo over the first few days, and then the release slowed, with roughly half of the cargo still encapsulated after 1 week. While the encapsulation efficiency of Rhodamine B depends on the length of the PLA chain, the release profile is similar for all block copolymers. The release profile of Nile Red, however, highly depends on the PLA chain length. PEGose_50_-PLA_100_ had a burst release on the first day, PEGose_50_-PLA_50_ had a linear sustained release, and PEGose_50_-PLA_25_ and PEGose_50_-PLA_15_ only released around 10% of their cargo. PEGose_50_-PLA_50_ nanoparticles seem to be the most efficient drug carriers, with the best encapsulation efficiency of both dyes and the most sustained release. The low leakage of PEGose_50_-PLA_25_ and PEGose_50_-PLA_15_ might also be promising if stimuli responsiveness is added to the drug delivery system. Additional functionalization of PEGose_50_-PLA_50_ might also be introduced to accelerate the release of hydrophobic compounds. Furthermore, other release media could be tested in the future, e.g., more acidic medium or phosphate buffer. Alternatively, poly(lactic-*co*-glycolic acid) could be used instead of PLA as a more easily degradable alternative.^66^

### Cytotoxicity and Cell Permeability

**Figure 6:**
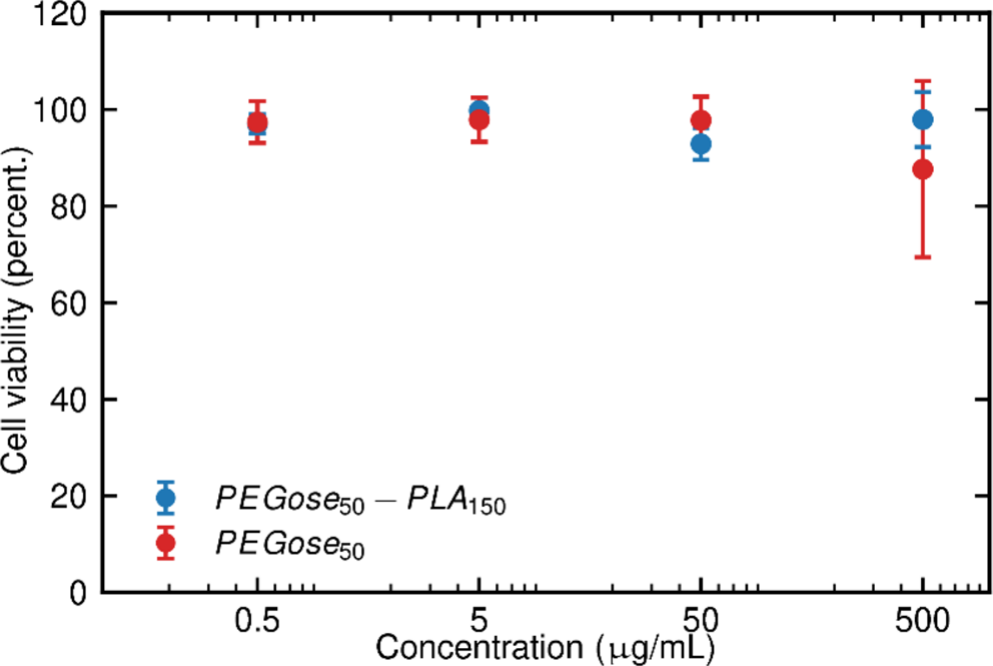
Human hepatocellular carcinoma cell viability after incubation with osmium-free PEGose_50_ or PEGose_50_-PLA_150_ nanoparticles prepared using the OsO_4_ dihydroxylation route with a concentration of 0.5, 5, 50, or 500 μg/mL in a humidified incubator at 37 °C under 5% CO_2_.

To assess the capacity of PEGose-PLA nanoparticles to be used in drug delivery, their cytotoxicity was evaluated. Cells from the human hepatocellular carcinoma cell line were chosen as a model system without a focus on a specific target in the body. Other systems will be elaborated in upcoming studies. The cells were cultured and then incubated with PEGose-PLA nanoparticles or PEGose homopolymers for 24 h at 37 °C. The AlamarBlue Cell Viability Reagent was then added as a cell health indicator. The fluorescence of cells incubated with nanoparticles or homopolymers was compared to a control without polymer to obtain a cell viability percentage (see Figure 6). Given that PEG and amylose are biocompatible, we expected that PEGose, which shares a similar structure, would be nontoxic to cells. *In vitro* cytotoxicity assays confirmed this hypothesis. Even with the highest concentration of 500 μg/mL, cell viability remained above 80% for PEGose and the nanoparticles. Interestingly, the cell viability with PEGose-PLA nanoparticles was higher than that with the PEGose homopolymer for the highest concentration tested. However, the cell viability values obtained at a 500 μg/mL concentration have a relatively high standard deviation: 18.2% for PEGose_50_ and 5.7% for the nanoparticles. This makes a direct comparison of the homopolymer and the nanoparticles difficult. The PEGose-PLA block copolymer was synthesized through the osmium dihydroxylation route; the very low amount used and the intensive dialysis steps seemed to have prevented any undesirable cytotoxicity. Cell viability when using the PEGose homopolymer decreased when increasing the concentration from 50 to 500 μg/mL, while the standard deviation considerably increased. Synthesizing the PEGose homopolymer through the osmium-free pathway did not seem to avert the drop in cell viability (see Figure 6).

**Figure 7:**
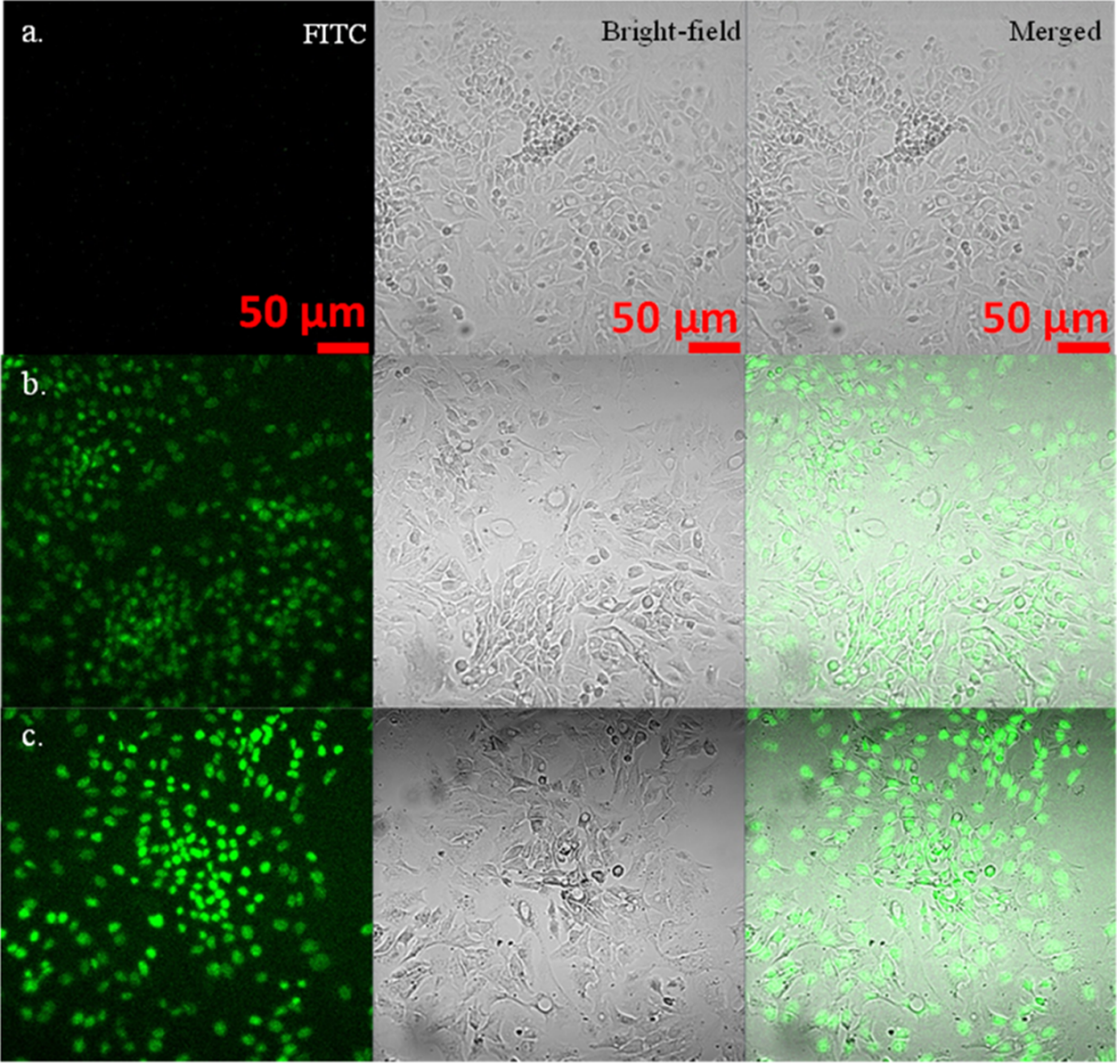
Confocal microscope images of (a) control (cells incubated without any polymer), (b) cells incubated with PEGose homopolymer, and (c) cells incubated with PEGose_50_-PLA_50_ nanoparticles. For each sample, the fluorescence, bright-field, and merged images are shown. Human hepatocellular carcinoma cells (2 × 10^4^ cells per well) were incubated at 37 °C in a 5% CO_2_ atmosphere for 24 h, with a polymer or nanoparticle concentration of 50 μg/mL.

To further explore the potential of PEGose-PLA nanoparticles as drug delivery systems, we studied their cell permeability. Fluorescein isothiocyanate (FITC) was chosen as the fluorescent label. A calibration curve with different solutions of FITC was prepared to determine the degree of substitution of the PEGose chains. The degree of substitution was very low (d.s. ≃ 0.0005 FITC units per PEGose repeating unit, meaning that roughly 2.5% of PEGose_50_ chains have a FITC tag) (see Figure S2), which suggests that the modification of the polymer structure was small enough to not affect the polymer cell permeability properties. After being incubated for 24 h at 37 °C, cells and polymers were observed under a confocal microscope under two different channels: one to inspect the fluorescence from the homopolymers or nanoparticles and the other one to obtain bright-field images of the cells. Merging the images from the two channels shows that the fluorescent spots from the PEGose homopolymer and PEGose-PLA nanoparticles superimpose with the cells observed through the bright-field channel (see Figure 7). These results indicate that both the homopolymer and nanoparticles were able to penetrate the cell membrane. The negligible cytotoxicity of the nanoparticles as well as their capability to cross the cell membrane exhibit their potential for intracellular drug delivery and further studies in the future.

## Conclusions

PEGose, a novel polymer that shares structural similarities with amylose and PEG, has been used to form a diblock copolymer with PLA. The polymer self-assembled into nanoparticles that were capable of encapsulating both hydrophilic and hydrophobic dyes. The low cytotoxicity, high cell permeability, and high stability of these drug carriers could lead to efficient cargo delivery in previously problematic harsh environments, such as the gastrointestinal tract. While additional *in vivo* biological tests need to be realized, the nanoparticles developed herein appear as a potential new avenue for drug delivery systems.

## Supporting Information Available

Additional analytical data (SEC, NMR, TEM, metal content) (PDF)
