# Supplementary material for: PEGose Block Poly(lactic acid) Nanoparticles for Cargo Delivery
Source: Macromolecules. 2024 Jun 14;57(13):6013–23. doi: 10.1021/acs.macromol.4c00528 (PMC11238580; doi:10.1021/acs.macromol.4c00528)
Supplement: Supplementary file 1 — ma4c00528_si_001.pdf [file ma4c00528_si_001.pdf]

Supporting Information

PEGose-*block*-poly(lactic acid) nanoparticles for cargo delivery

Jean-Baptiste Masclef,[a] *Emmanuelle M. N. Acs,*[a] *Jesko Koehnke,*[a,b] *Joëlle Prunet,*[a] *Bernhard V. K. J. Schmidt*[a]

[a] School of Chemistry, University of Glasgow, Joseph Black Building, G12 8QQ, Glasgow, UK

[b] Institute of Food Chemistry, Leibniz University Hannover, 30167 Hannover, Germany

Email: joelle.prunet@glasgow.ac.uk; bernhard.schmidt@glasgow.ac.uk

Table S1. Residual ruthenium content determined by ICP-MS after various purification methods: addition of DMSO followed by filtration, gel permeation chromatography with Sephadex LH-20, CupriSorb heavy metals resin, oxidative procedure with H_2O_2.

**Purification procedure**	**Purification efficiency**	**Ruthenium content (ppm)**	**RSD%**
Unpurified	0%	5941	0.79
DMSO, filtration	74.0%	1544	0.89
DMSO, filtration, GPC	79.3%	1231	1.31
DMSO, filtration, GPC, CupriSorb	92.0%	475	1.24
Oxidative procedure	99.5%	32	2.39

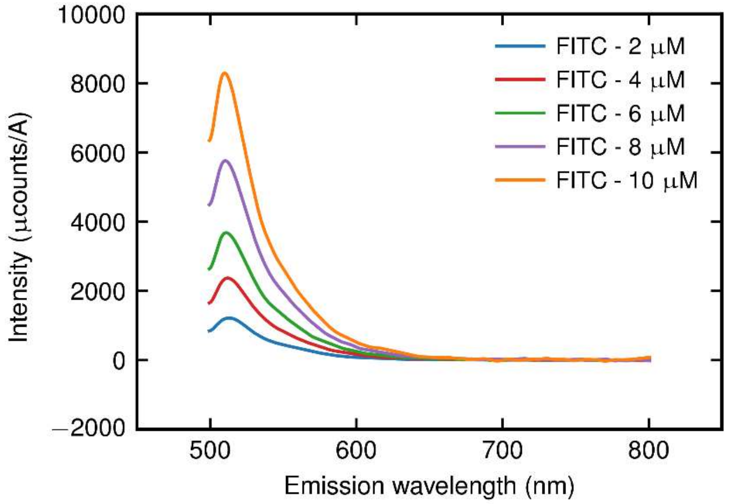

Figure S1. Fluorescence trace of FITC solutions in a Tris buffer with concentration ranging from 2 μM to 10 μM.

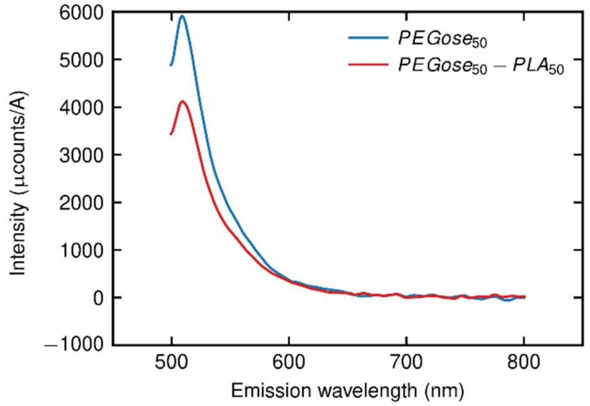

Figure S2. Fluorescence trace of FITC-labelled PEGose$_{50}$ and PEGose$_{50}$-PLA$_{50}$.

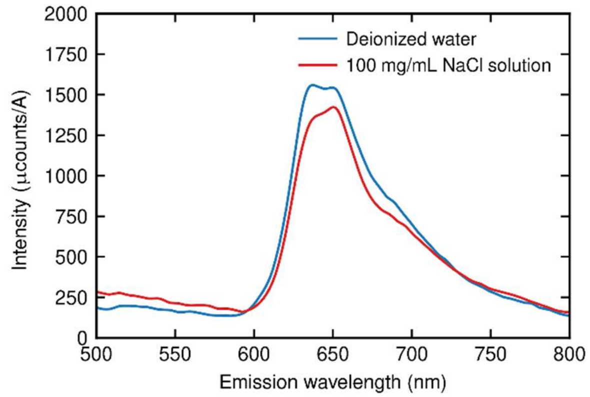

Figure S3. Fluorescence trace of PEGose$_{50}$-PLA$_{50}$ nanoparticles loaded with Nile Red in deionized water and in a 100 mg/mL NaCl solution

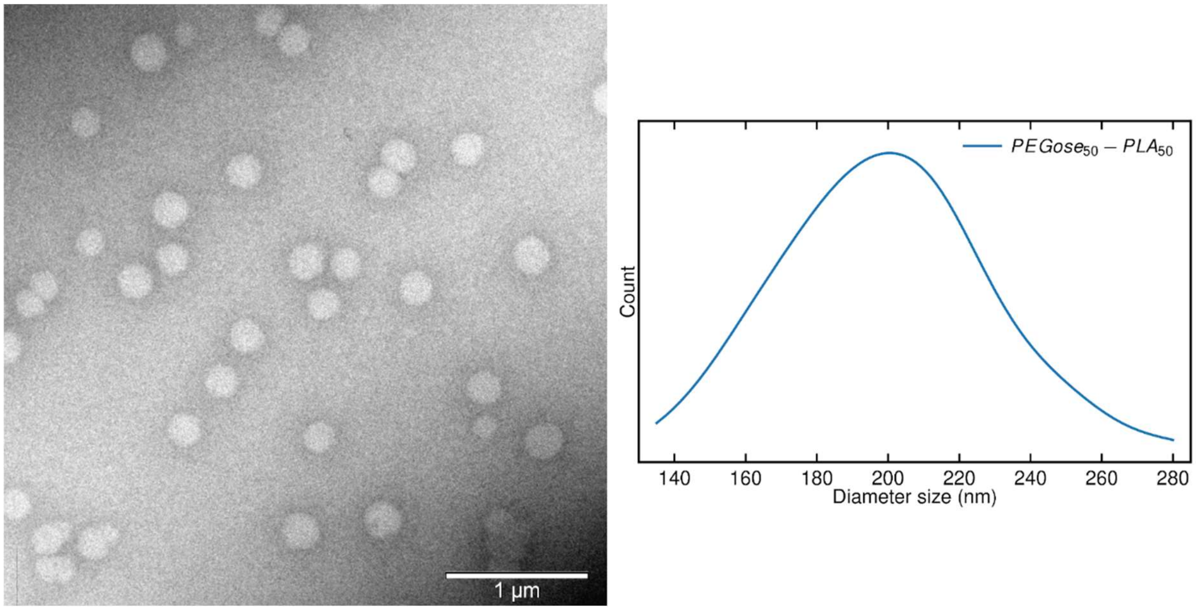

Figure S4. TEM image of PEGose$_{50}$-PLA$_{50}$ nanoparticles and the corresponding size distribution.

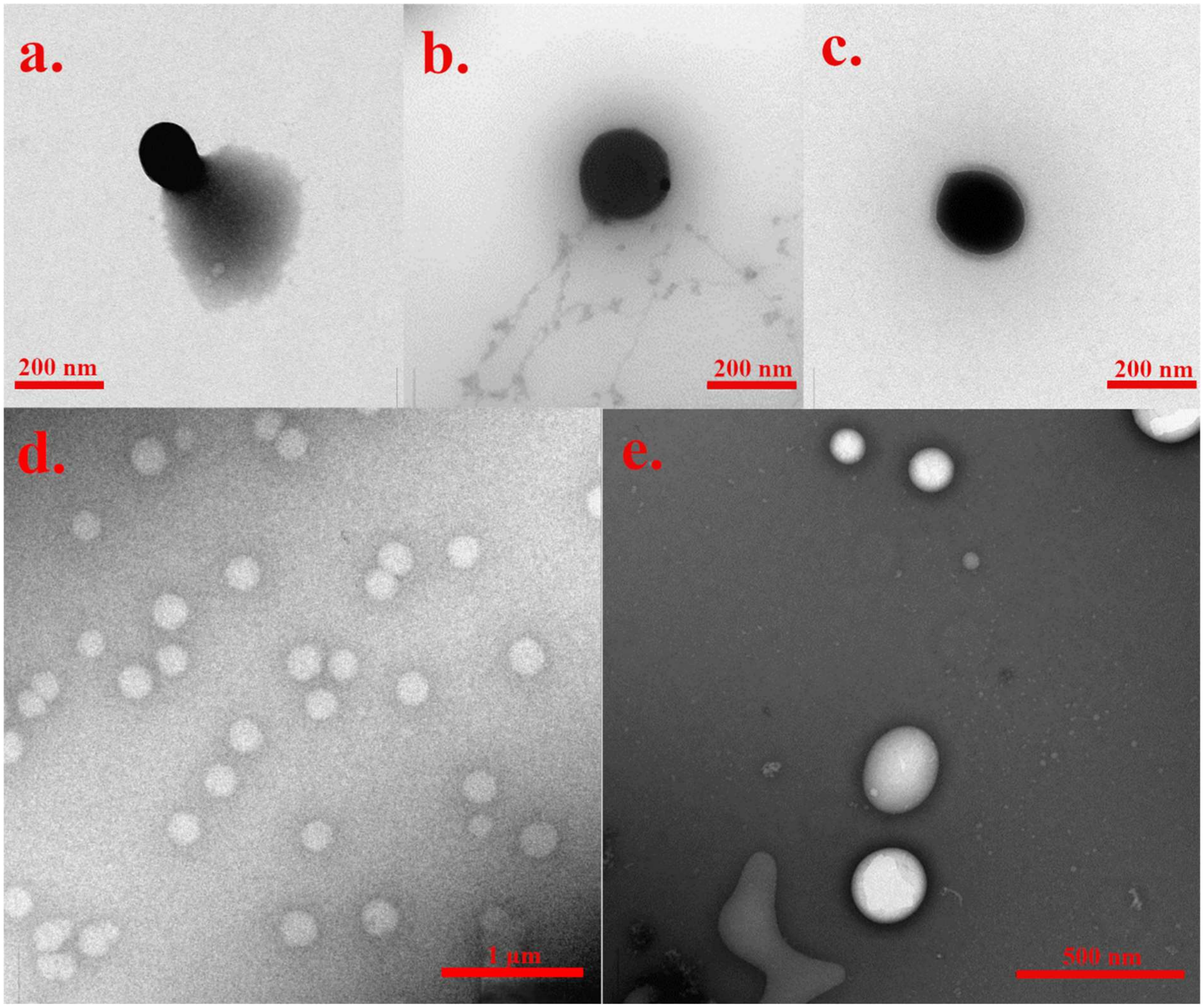

Figure S5. TEM images of PEGose$_{50}$-PLA$_{50}$ stained with uranyl acetate (a. b. and c.) or without using any stain (d. and e.). The nanoparticles were prepared by dialyzing the DMSO block copolymer solution against deionized water.

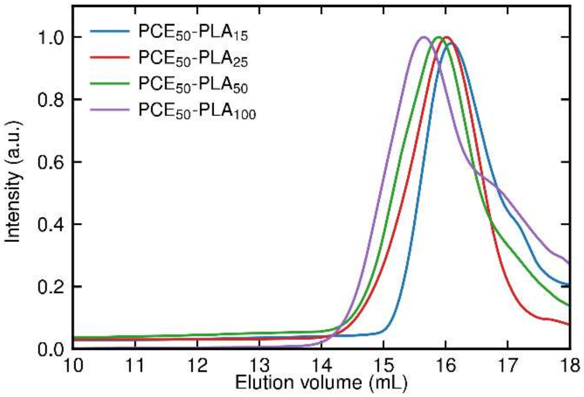

Figure S6. SEC traces of PCE$_{50}$-PLA$_{15}$, PCE$_{50}$-PLA$_{25}$, PCE$_{50}$-PLA$_{50}$ and PCE$_{50}$-PLA$_{100}$ measured in THF with a 1 mL/min flow rate.

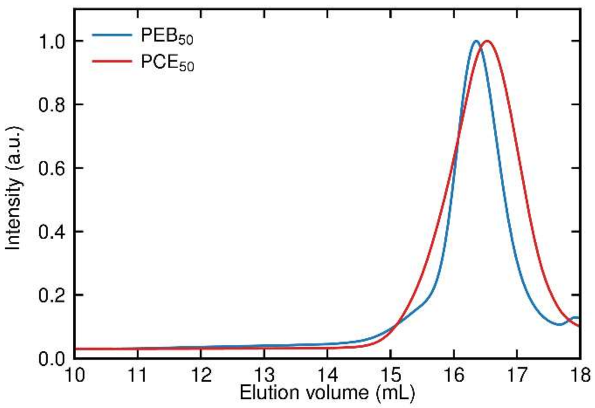

Figure S7. SEC traces of PEB_{50} and PCE_{50}, measured in THF with a 1 mL/min flow rate.

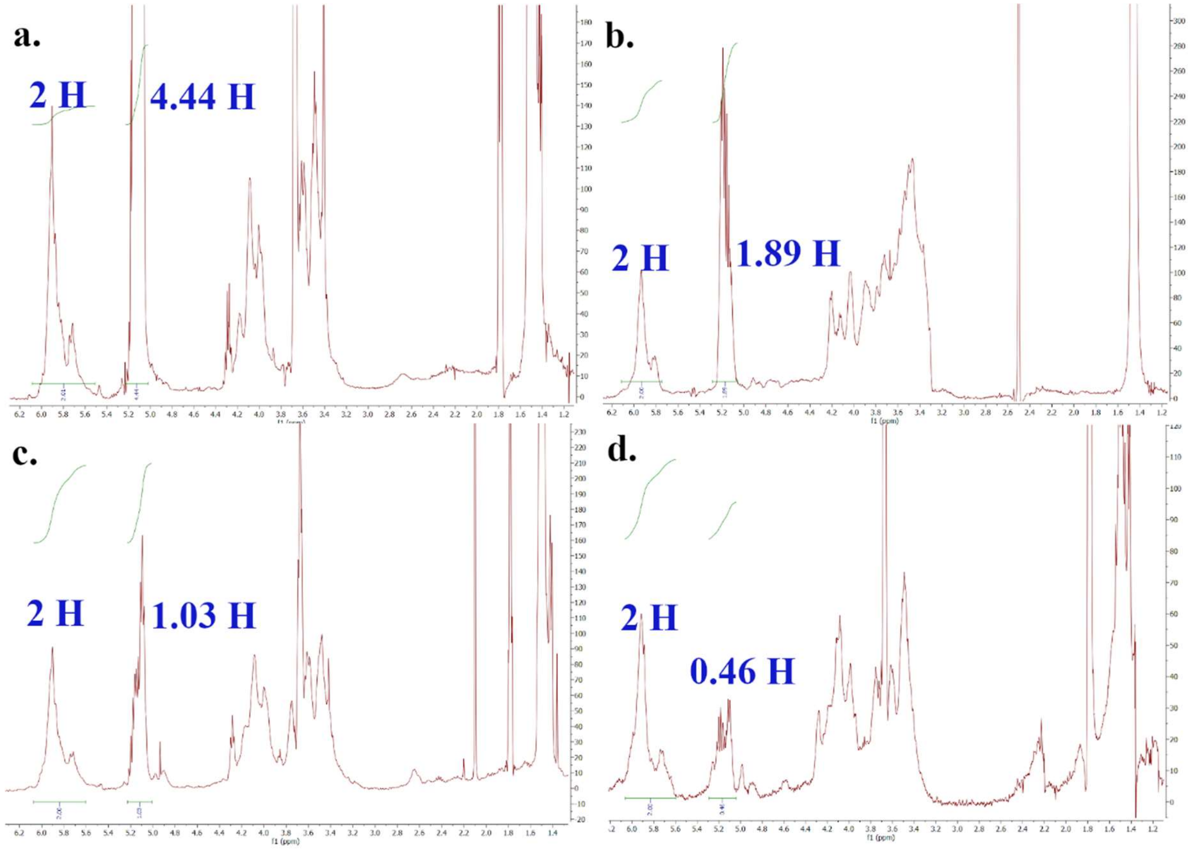

Figure S8. ^{1}H NMR spectra of **a)** PCE_{50}-PLA_{100}, **b)** PCE_{50}-PLA_{50}, **c)** PCE_{50}-PLA_{25} and **d)** PCE_{50}-PLA_{15} in $CDCl_3$. Alkene protons from PCE were normalized to 2.

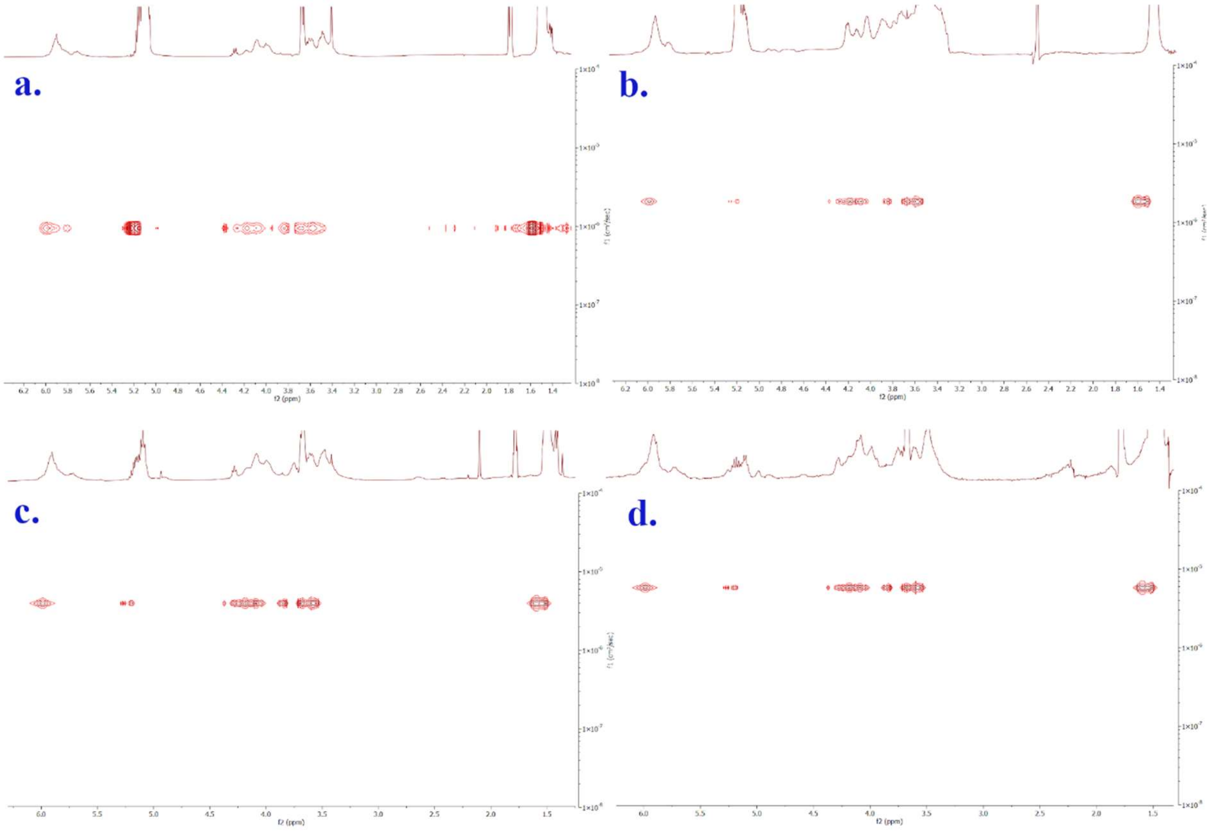

Figure S9. ^{1}H NMR DOSY spectra of **a)** PCE_{50}-PLA_{100}, **b)** PCE_{50}-PLA_{50}, **c)** PCE_{50}-PLA_{25} and **d)** PCE_{50}-PLA_{15} in $CDCl_3$.

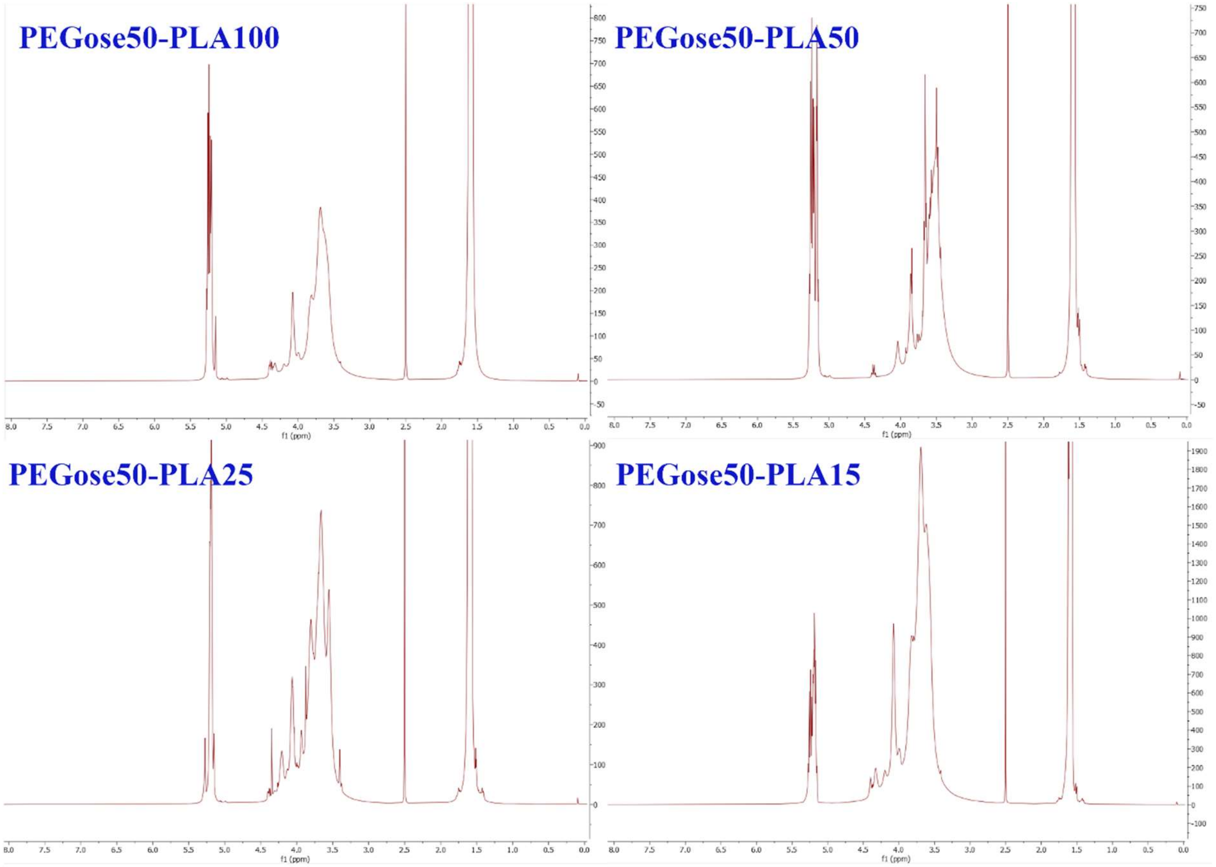

Figure S10. ^{1}H NMR spectra of $PEGose_{50}$-PLA_{100}, $PEGose_{50}$-PLA_{50}, $PEGose_{50}$-PLA_{25} and $PEGose_{50}$-PLA_{15} in DMSO-d_6.

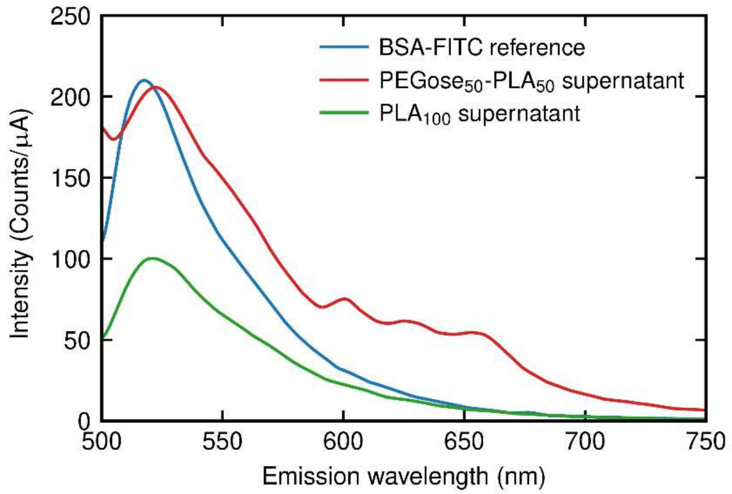

Figure S11. Fluorescence trace of a fluorescein isothiocyanate (FITC) labelled bovine serum albumin (BSA) protein. Fluorescence trace of $PEGose_{50}$-*b*-PLA_{50} and PLA_{100} nanoparticles after incubation with FITC-labelled BSA for 24 h, followed by centrifugation.
